# Investigation of genetic factors regulating chlorophyll and carotenoid biosynthesis in red pepper fruit

**DOI:** 10.3389/fpls.2022.922963

**Published:** 2022-09-15

**Authors:** Siyoung Jang, Geon Woo Kim, Koeun Han, Young Min Kim, Jinkwan Jo, Seo-Young Lee, Jin-Kyung Kwon, Byoung-Cheorl Kang

**Affiliations:** Department of Agriculture, Forestry and Bioresources, Research Institute of Agriculture and Life Sciences, Plant Genomics and Breeding Institute, College of Agriculture and Life Sciences, Seoul National University, Seoul, South Korea

**Keywords:** fruit color intensity, plastid conversion, chlorophylls, carotenoids, quantitative trait locus (QTL), *Capsicum annuum* (Pepper)

## Abstract

Chlorophylls and carotenoids are synthesized in the chloroplast and chromoplast, respectively. Even though the two pigments are generated from the same precursor, the genetic correlation between chlorophyll and carotenoid biosynthesis has not yet been fully understood. We investigated the genetic correlation of chlorophyll and carotenoid biosynthesis during fruit ripening. Two recombinant inbred lines populations, “Long Sweet” × “AC2212” (“LA”) RILs derived from a cross between *Capsicum annuum* “Long Sweet” with light-green and light-red fruit and *C. annuum* “AC2212” with dark-green and brown-fruit and “3501 (F)” × “3509 (C)” (“FC”) RILs from *C. annuum* “3501” with dark-green and dark-red fruit and *C. annuum* “3509” with intermediate green and light-red fruit, were used. As the fruit ripened, three accessions produced high levels of xanthophyll. The dark-green immature fruit accumulated more total carotenoids than the light-green fruit. This trend corresponded to the expression pattern of *1-deoxy-d-xylulose 5-phosphate synthase* (*DXS*) and *CaGLK2* genes during fruit development. The expression levels of *DXS* and *CaGLK2* in the dark-green accession “3501” were significantly higher than those of “3509” and “Long Sweet” during the early stages of fruit development. Furthermore, the genotype analysis of the transcription factor controlling chloroplast development (*CaGLK2*) in LA RILs revealed that *CaGLK2* expression affected both carotenoid and chlorophyll contents. The single nucleotide polymorphism (SNP) linkage maps were constructed using genotyping-by-sequencing (GBS) for the two populations, and QTL analysis was performed for green fruit color intensity and carotenoid content. The QTL (*LA_BG-CST10*) for capsanthin content in LA RILs located at 24.4 to 100.4 Mbp on chromosome 10 was overlapped with the QTL (*FC15-Cap10*) for capsanthin content in FC RILs. Three QTLs for capsanthin content, American spice trade association (ASTA) value, and immature green fruit color intensity were also overlapped from 178.2 to 204 Mbp on chromosome 10. At the location, 151.6 to 165 Mbp on chromosome 8, QTLs (*FC15-tcar8, FC17-ASTA8.1, and FC17-ASTA8.2*) for total carotenoid content and ASTA value were discovered, and this region contained *2-C-methyl-d-erythritol 4-phosphate cytidylyltransferase* (*MCT*), which is involved in the MEP pathway. This result is the first report to show the correlation between carotenoid and chlorophyll biosynthesis in pepper. This research will expand our understanding of the mechanism of the chloroplast-to-chromoplast transition and the development of high pigment pepper varieties.

## Introduction

Humans have domesticated colorful crops due to the ornamental, nutritional, and medical values of color pigments. The fruit of the solanaceous plants changes its color as the fruit develops from immature to mature stages. Immature color varies from ivory to dark green, which changes to yellow, orange, red, and brown as the fruit ripens. The color of the mature fruit is determined by the varying amounts of carotenoids, chlorophylls, flavonoids, and anthocyanin.

Immature pepper fruit color is usually green because chlorophylls are major pigments. Chlorophylls, comprised of chlorophyll *a* and *b*, are synthesized and stored in the chloroplast. The major role of these pigments is the delivery of electrons for converting light to chemical energy (Hashimoto et al., 2016). Chlorophyll biosynthesis can be divided into three steps: (i) synthesis of chlorophyll *a* from glutamate, (ii) interconversion between chlorophyll *a* and chlorophyll *b*, and (iii) degradation of chlorophyll *a* (Tanaka and Tanaka, 2006). Chloroplast is converted to chromoplast from the breaker stage fruit (Bian et al., 2011; Egea et al., 2011; Wise, 2016), which leads to either orange or red color of the mature fruit due to the rapid accumulation of carotenoids in the chromoplast. Carotenoids and chlorophylls are produced from the same precursor, geranylgeranyl diphosphate (GGPP), which is converted to phytoene by phytoene synthase (PSY). Carotene and lycopene are synthesized from phytoene, and lycopene is cyclized to form α-carotene and β-carotene. Finally, xanthophylls are generated by hydroxylation of carotenes (Nisar et al., 2015). Antheraxanthin and violaxanthin are converted to capsanthin and capsorubin by capsanthin–capsorubin synthase (CCS) in pepper (Guzman et al., 2010; Wahyuni et al., 2011). The composition of carotenoids in red pepper is diverse (Arimboor et al., 2015). During ripening, red pigments increase, but capsanthin and capsorubin play a significant role in red pepper fruit. In total, 30–70% of the carotenoid pigment capsanthin is present in red fruits.

Genetic research on chlorophyll synthesis in the Solanaceous crop was started from the green shoulder phenotype of mature tomato fruit (Powell et al., 2012). *SlGLK2* and *KNOX* genes are known to regulate chloroplast development in tomato fruit. The *Uniform ripening* (*U*) locus encoding *SlGLK2* on chromosome 10 determines the green shoulder of the tomato fruit. The functional *SlGLK2* allele results in the green shoulder phenotype. *GLK2* is differentially expressed and influenced the green coloration along a gradient from the stem end to the blossom end of the tomato fruit (Nguyen et al., 2014). The *uniform gray-green* (*ug*) locus also determines the green shoulder of the tomato, whereas the *Curl* (*cu*) mutation results in the increase of chlorophyll contents (Nadakuduti et al., 2014). The *ug* and *Cu* loci encode *TKN4* and *TKN2*, respectively, both of which encode *KNOTTED1-LIKE HOMEOBOX* (*KNOX*) transcription factors. *Arabidopsis Pseudo Response Regulator2* (APRR2)-like transcription factor was reported to have a correlation with the chlorophyll contents in immature fruit and carotenoid contents in mature fruit in tomatoes (Pan et al., 2013). In pepper, a genetic study was conducted to understand the controlling mechanism of the chlorophyll content in the whole fruit. Two major QTLs, *pc8.1* and *pc10.1*, were detected in an interspecific F_2_ population and NILs (Brand et al., 2012). Among the two QTLs, *pc10.1* on chromosome 10 turned out to be an *SlGLK2* homolog, *CaGLK2*, annotated as MGMT_Contig23879 (Brand et al., 2014). An ortholog of *SlAPRR2* was also related to pigment accumulation in sweet pepper (Pan et al., 2013). *pc8.1* located on chromosome 1 encodes *zinc-finger transcription factor* LOL1 (*LSD ONE LIKE1*; *CcLOL1*) (Borovsky et al., 2019).

The color of mature pepper fruit ranges from ivory to dark red depending on carotenoid composition. Hurtado-Hernandez and Smith (1985) suggested the three-loci model for mature pepper fruit color. The red color trait is dominant over yellow and orange, and two loci were determined to encode *CCS* and *PSY1* by genetic mapping (Lefebvre et al., 1998; Huh et al., 2001). The third locus, *c1*, encodes the pseudo response regulator 2-like protein PRR2 (Jeong et al., 2020; Lee et al., 2020). In addition, white fruit color is determined by mutations in all three genes [*CCS*, *PSY1*, and *PRR2;* Jeong et al. (2020)]. However, *PSY1*, *CCS*, and *PRR2* of the three-loci model cannot explain all variations in fruit color. Jeong et al. (2019) compared the carotenoid composition of 43 accessions and revealed the allelic diversity of six genes (*PSY1, PSY2, Lcyb, CCS, CrtZ*, and *ZEP*). *PSY2* also participates in carotenoid metabolism when *PSY1* is absent (Jang et al., 2020). However, *PSY1*, *CCS*, and *APRR2* of the three-loci model cannot explain all fruit color variations. The carotenoid composition in orange-colored fruit is caused by the complementary synthesis of β-carotene and capsanthin (Guzman et al., 2010), implying that β-carotene hydroxylase (*CrtZ2*) controls β-carotene contents (Borovsky et al., 2013), and zeaxanthin is converted to antheraxanthin and violaxanthin by zeaxanthin epoxidase (ZEP) (Lee et al., 2021). In contrast to an abundance of research on color determining genes, QTL studies on carotenoid contents are rare. The expression levels of *1-deoxy-d-xylulose 5-phosphate synthase* (*DXS*) and *phytoene synthase-1* (*PSY-1*) are related to capsanthin content, which brings on various red color intensities (Berry et al., 2019). In addition, two major QTLs for capsanthin contents were detected on chromosomes 6 and 9 (Konishi et al., 2019).

In this study, we examined the genetic factors that regulate the biosynthesis of chlorophyll and carotenoids in red pepper. The chlorophyll and carotenoid accumulation profiles of various colored cultivars were analyzed. Analysis was conducted on the expression of carotenoid biosynthetic genes and chlorophyll development transcription factors. We demonstrated a correlation between the accumulation of carotenoids and the expression of *DXS* and *PSY1* in red pepper. The genetic analysis of two RIL populations revealed a correlation between genes involved in chlorophyll biosynthesis and the accumulation of carotenoids. The carotenoid content varied depending on the *CaGLK2* genotype. We performed a QTL analysis on the color variation of immature and mature fruit in two RIL populations. The QTLs for capsanthin content, American spice trade association (ASTA) value, and intensity of green color in immature fruit were revealed.

## Materials and methods

### Plant materials

*Capsicum annuum* “Long Sweet” and *C. annuum* “AC2212” were crossed to develop recombinant inbred lines (RILs). “Long Sweet” has light green immature fruits and red mature fruits, and “AC2212” has dark green immature fruits and brown mature fruits. F_7:9_ LA (“Long Sweet” ×”AC2212”) RILs were used to evaluate fruit color and genotyping-by-sequencing (GBS). F_7:9_ RILs were cultivated in a plastic house at the Seoul National University farm (Suwon, South Korea) in 2019. A total of 254 RILs were examined for ASTA value and green intensity, and 96 RILs were used to construct GBS libraries and carotenoid content analysis.

*Capsicum annuum* “3501” and *C. annuum* “3509”, which are provided by the National Institute of Horticultural and Herbal Science (Wanju, Korea), were also used to develop RILs. “3501” has a dark red mature fruit color and dark green immature fruit, and “3509” has a light red mature fruit color and light green immature fruit. FC (“3501” × “3509”) F_5:7_ RILs and F_7:9_ RILs were used to evaluate carotenoid contents, and F_6:8_ RILs were used to evaluate GBS. A total of 176 F_5:7_ RILs were cultivated in a plastic house at the Seoul National University farm in 2015, and F_7:9_ RILs were cultivated in a plastic house (Cheongju, South Korea).

The parental lines of *C. annuum* “Long Sweet,” “AC2212,” “3501,” and “3509” were examined in gene expression analysis, color pigment measurement, and transmission electron microscopy (TEM) imaging.

### Visual evaluation of green immature fruit color and American spice trade association color value analysis

The green intensity of fresh immature fruits was evaluated for 254 LA RILs. We divided green intensity into five classes (Supplementary Figure 1). The lightest green immature fruit color, like in “Long Sweet,” was referred to as class 1, and the darkest green immature fruit color like “AC2212” was referred to as class 5. Green immature fruits from three individual plants of 254 RILs were harvested and photographed against a white light background. The green intensity was cross-checked by two people.

The American spice trade association value was determined for 254 LA RILs and 176 FC RILs. Red mature fruits from three individual plants of 254 RILs were freeze-dried. About 0.1 g of pepper powder with 100 ml of acetone was incubated for around 16 h, and the absorbance at 460 nm was measured using a spectrophotometer (UV-Vis 2550, Shimadzu, Kyoto, Japan). ASTA value was calculated using the following equation:


$$\mathrm{ASTA}\,\mathrm{value}=16.4\times \frac{{\text{A}}_{460}}{\mathrm{pepper}\,\mathrm{powder}\,\mathrm{weight}\,\left(g\right)}$$

### Chlorophyll extraction and measurements

Chlorophyll contents were measured in the four accessions (“AC2212,” “Long Sweet,” “3501,” and “3509”) as followed by Lichtenthaler and Buschmann (2001) and Yoo et al. (2013). Fruit pericarps from three individual plants were harvested at fruit developmental stages (10 and 20 days after fruit set, mature green, breaker, 5 and 10 days after breaker, fully red). Pericarps were freeze-dried and pooled. Chlorophylls were leached from 0.5 g of dried pepper powder into a total of 25 ml of high-performance liquid chromatography (HPLC)-grade methanol. Absorbance at 665 and 652 nm for chlorophyll *a*, and absorbance at 645 and 662 nm for chlorophyll *b* were measured using a spectrophotometer (UV-Vis Vis 2550, Shimadzu, Kyoto, Japan). Chlorophyll *a* and *b* were calculated by Lichtenthaler and Buschmann equations. Total chlorophyll contents were calculated by adding up two chlorophylls.


$$\mathrm{Chlorophyll}\,a\,\left(\mu \,\text{g}/\text{ml}\right)=16.72\times A_{665}-9.16\times A_{652}$$

$$\mathrm{Chlorophyll}\,b\,\left(\mu \,\text{g}/\text{ml}\right)=20.13\times A_{645}-4.19\times A_{662}$$

### Carotenoid pigments extraction and high-performance liquid chromatography analysis

Carotenoid pigments were extracted from 96 LA RILs and 176 FC RILs as described by Lee et al. (2021) with some modifications. Pepper powder was pooled from three individual plants and 0.05 g of pooled powder was used to extract carotenoids. Samples were dissolved in 500 μl of HPLC-grade acetone (Honeywell, Charlotte, NC, United States) and filtered using a 0.2-μm syringe filter (Acrodisc LC 13-mm syringe filter, PVDF membrane, Pall, NY, United States). The HPLC was performed using Ultimate3000 HPLC (Thermo Dionex, Sunnyvale, CA, United States) at the National Instrumentation Center for Environmental Management (Seoul, South Korea). We analyzed seven carotenoids, capsanthin, capsorubin, lutein, zeaxanthin, β-cryptoxanthin, β-carotene, and α-carotene (CaroteNature, Münsingen, Switzerland), as standards.

Carotenoid pigments were extracted from 176 FC RILs. The HPLC was performed using Ultimate3000 HPLC (Thermo Dionex, Sunnyvale, CA, United States) in the Molecular Marker and Food Ingredients Analysis Center of Agricultural Science Research Institute at Chungnam National University.

### Genomic DNA isolation and genotyping by sequencing library construction

Genomic DNA was extracted from small young leaves of 254 LA RILs and 176 FC RILs by the CTAB method as described previously (Lee et al., 2020). Young leaves were prepared in a 1.4-ml tube of Racks for Matrix™ 2D Barcoded Storage Tubes (Thermo Scientific™, United States). Genomic DNA (gDNA) was diluted to 80 ng/μl in distilled water. GBS libraries were constructed as follows by Han et al. (2018). About 4,000 ng of LA RILs gDNA were digested with two restriction enzymes, *EcoR*I/*Mse*I, and the gDNA of FC RILs was digested with PstI/*Mse*I. The digested DNA was ligated to adapters and amplified with universal primer sets for Illumina sequencing. GBS libraries generated from 96 adapters with six nucleotide barcodes were pooled in three tubes and sequenced in three lanes using Hiseq X (Illumina, San Diego, CA) at Macrogen (Seoul, South Korea).

### Single nucleotide polymorphism calling and genetic map construction

Single nucleotide polymorphisms were carried out as described by Han et al. (2018) with some modifications. Sequencing reads with the barcode were trimmed and demultiplexed by CLC Genomics Workbench software (version 8.5.1). Raw 151 bp reads of libraries were trimmed with a minimum length of 80 bp and a minimum quality of Q20 and demultiplexed by each barcode. The filtered reads were aligned to the *C. annuum* Dempsey reference genome v.1.0 using the Burrows-Wheeler Aligner program v0.7.17 (Li and Durbin, 2010). Single nucleotide polymorphism (SNP) variants were analyzed by the Genome Analysis Tool Kit (GATK) (DePristo et al., 2011) v4.1.7.0 HaplotypeCaller. Raw SNPs were filtered with the following criteria: QUAL > 30, DP > 3, AF > 1.00, AC < 1 by GATK v4.1.7.0 VariantFiltration. SNPs between parental lines were selected. Finally, if the calling rate was lower than 10%, the SNPs were eliminated. Missing genotypes were imputed and phased as described by Hong et al. (2020) using BEAGLE through the R package “synbreed” (Wimmer et al., 2012).

Genetic maps constructed using CarthaGene software v1.3. SNP set was mapped with a logarithm of the odds (LOD) threshold of 3.0 and a distance threshold of 50 cM. The Kosambi mapping function was used to calculate the genetic distance between SNPs. Genetic and physical maps of the reference genome and Quantitative trait loci (QTLs) were drawn by MapChart software v.2.32 (Voorrips, 2002).

### Quantitative trait locus analysis for carotenoid contents and green fruit color

Quantitative trait loci for carotenoid contents and green fruit color were analyzed using LA genetic map and ten traits (capsanthin, capsorubin, lutein, zeaxanthin, β-cryptoxanthin, β-carotene, α-carotene, total carotenoid contents, ASTA value, and green fruit color intensity). Seven carotenoid contents were used as described in Section “Visual evaluation of green immature fruit color and American spice trade association color value analysis,” and total carotenoid contents were calculated by adding seven carotenoid contents. In addition, green intensity and ASTA value were used to detect QTLs. QTLs for carotenoid contents in FC RIL were analyzed using an FC genetic map and five traits (capsanthin, capsorubin, β-carotene, total carotenoid contents, and ASTA value). The total carotenoid contents of FC RILs were calculated by adding up three carotenoid contents.

Composite interval mapping (CIM) was analyzed to detect QTLs as described by Park et al. (2019) using Windows QTL Cartographer software 2.5 (Wang et al., 2012). The LOD threshold was determined *via* 1,000 permutations with a 5% probability.

### RNA isolation, cDNA synthesis, and gene expression analysis by real-time qRT-PCR

Total RNA was extracted from four accessions (“Long Sweet,” “AC2212,” “3501,” and “3509”) using the MG Total RNA Extraction Kit (MGmed, South Korea) as described by Lee et al. (2021). Fruit pericarp samples from three individual plants were prepared at seven fruit developmental stages (10 days after fruit set, 20 days after fruit set, mature green, breaker, 5 days after breaker, 10 days after breaker, and fully red [FR]). cDNA was synthesized from 1.5 μg of total RNA by TransScript^®^ All-in-one First-Strand cDNA Synthesis SuperMix for qPCR (Transgen Biotech, China).

The expression of seven genes including four carotenoid-related genes, *PSY1*, *CCS*, *APRR2*, and *DXS*, and three chlorophyll-related genes, *CaGLK2*, *CcLOL1*, and *SGR* were analyzed. Primer information for PCR amplification is described in Supplementary Table 1. The *actin* gene was used as an internal control for qRT-PCR. Primer sequences were as follows: (forward) 5′- ATCCCTCCACCTCTTCACTCTC-3′, (reverse) 5′- GCCTTAACCATTCCTGTTCCATTATC-3′.

### Transmission electron microscopy analysis

Transmission electron microscopy was conducted to analyze plastid development using samples at three stages, mature green, breaker, and mature red, of fruit pericarp of the four accessions. Specimens were prepared as described by Lee et al. (2021). Polymerized samples were trimmed and sliced to a thickness of 80 nm, and the plastids were observed under a JEM-1400 Flash (JEOL, Tokyo, Japan) (120 kV) TEM by Dental Research Institute (Seoul National University, Seoul).

### Genotype analysis of *CaGLK2* and *APRR2* in LA recombinant inbred lines

*CaGLK2* and *APRR2* partial sequences from “Long Sweet” and “AC2212” were analyzed for polymorphism and the development of molecular markers. In *CaGLK2*, the substitution of cytosine (C) for thymine (T) resulted in a synonymous mutation in the third exon, and an HRM marker was designed based on this SNP. The sequence of the primer set was as follows: CaGLK2-HRM(F) contains the sequence “5′-TGGACCCAAAGGAAGCAAAT-3′” and CaGLK2-HRM(R) contains the sequence “5′-TGCCCCCAAACATGTAAGG-3′.” To analyze the HRM markers, 60 ng of DNA, 10 X Hifi buffer, 2.5 mM dNTPs, 10 pmol/μl of each primer, 1 unit of *Taq* polymerase, and 0.6 μl of SYTO9 were added to the reaction mixture. The PCR conditions were as follows; 95°C for 4 min, followed by 55 cycles of 95°C for 10 s, 60°C for 20 s, and 72°C for 20 s, and a final extension at 72°C for 10 min. For the HRM analysis, one cycle was added at the end. The temperature was increased by 9.1°C per second from 65 to 90°C to dissociate the double-stranded DNA with SYTO9 into a single-stranded DNA. In the eighth intron of APRR2, adenine (A) was replaced by guanine (G). The CAPS marker was developed using this SNP’s *Sau96* I restriction site. The annealing temperature and elongation time for the PCR step were 60°C and 50 s, respectively. The PCR products were digested for 4 h at 37°C with the enzyme *Sau96* I. On a 1.2% agarose gel, the digested DNA was loaded.

## Results

### Chlorophyll and carotenoid accumulation profiles in parental lines

**FIGURE 1:**
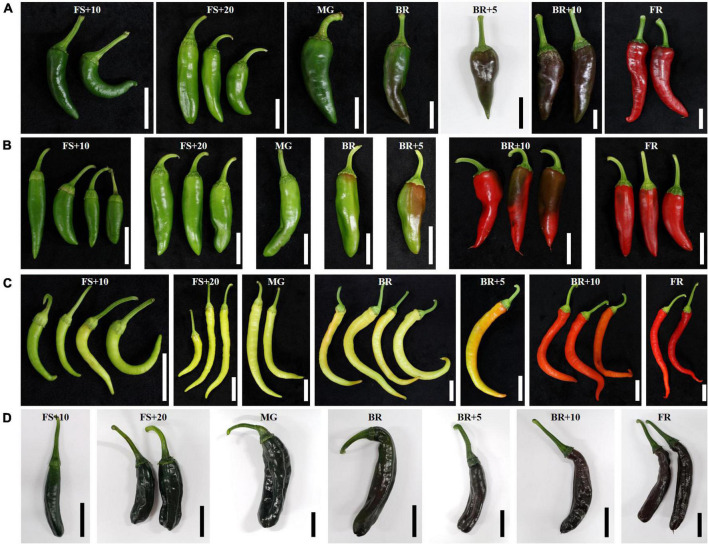
Fruit color change during ripening of **(A)** “3501,” **(B)** “3509,” **(C)** “Long Sweet,” and **(D)** “AC2212.” FS, fruit set, MG, mature green, BK, breaker stage, FR, full red color fruit. White and black bars indicate 3 cm.

The fruit color phenotypes of “AC2212,” “Long Sweet,” “3501,” and “3509” were diverse (Figure 1). Immature fruits from “Long Sweet” were light green and ripened to a light red color, while those from “3509” were an intermediate green and changed to intermediate red. The immature fruits of “3501” and “AC2212” were dark green in color, and “3501” turned dark red, while “AC2212” turned brown.

To determine the color pigment profile during fruit ripening, two chlorophylls and seven carotenoids were analyzed (Table 1). Chlorophyll content patterns varied between four accessions, but total chlorophyll content decreased overall following the breaker stage that initiated carotenoid accumulation. “AC2212” had the highest concentration (73–86 μg/ml), followed by “3501” (33–61 μg/ml), “3509” (24–55 μg/ml), and “Long Sweet” (23–40 μg/ml).

**TABLE 1:** Carotenoid contents of four accessions throughout fruit development and ripening.

Accessions	Stages	Chlorophyll contents (μg/ml)			Carotenoid contents (μg/g DW)							
			
		Chlorophyll *a*	Chlorophyll *b*	Total chlorophylls	Capsanthin	Capsorubin	Lutein	Zeaxanthin	β -cryptoxanthin	β -carotene	α -carotene	Total carotenoids
3501	FS+10	30.91	22.86	53.77	-	-	162.7	1.2	-	62.8	-	226.6
	FS+20	29.12	31.85	60.97	-	-	91.4	1.3	-	34.9	-	127.6
	MG	30.07	31.27	61.34	-	-	115.9	9.7	-	43.6	7.6	176.8
	BR	27.84	33.25	61.09	69.6	-	56.4	51.9	-	48.3	-	226.3
	BR+5	23.53	20.99	44.51	152.1	-	31.3	92.4	9.4	-	-	285.2
	BR+10	21.99	25.30	47.30	439.6	18.6	30.9	338.4	44.0	-	-	871.4
	FR	11.54	21.68	33.21	794.9	22.7	50.8	239.4	31.4	134.5	-	1,273.8
3509	FS+10	22.18	16.34	38.52	-	-	100.7	1.0	-	29.9	-	131.6
	FS+20	26.21	29.00	55.21	-	-	73.7	1.2	-	24.7	-	99.7
	MG	18.45	22.71	41.16	-	-	51.5	2.8	-	14.3	-	68.6
	BR	21.36	22.91	44.28	36.0	-	35.1	15.7	-	24.5	-	111.2
	BR+5	21.89	32.06	53.95	46.8	-	19.3	29.7	-	-	-	95.8
	BR+10	9.52	16.94	26.46	227.2	9.7	17.8	84.7	14.6	-	-	354.0
	FR	7.94	16.26	24.20	304.7	7.1	13.7	48.4	14.8	-	-	388.7
Long Sweet	FS+10	14.27	17.31	31.58	-	-	55.1	0.3	-	19.4	-	74.8
	FS+20	14.68	25.79	40.47	-	-	15.9	0.6	-	-	-	16.5
	MG	8.53	14.39	22.92	-	-	12.8	0.3	-	-	-	13.2
	BR	10.51	20.61	31.11	-	-	4.4	0.6	-	-	-	5.0
	BR+5	12.30	23.04	35.34	21.5	-	5.0	2.9	-	-	-	29.3
	BR+10	10.88	21.05	31.92	93.2	-	8.0	15.4	-	-	-	116.6
	FR	10.86	23.13	33.99	185.1	-	21.9	98.4	77.7	138.1	-	521.2
AC2212	FS+10	28.55	56.38	84.93	-	-	574.3	-	83.5	216.1	109.1	983.1
	FS+20	25.03	51.84	76.87	-	-	428.3	-	60.0	173.5	81.0	742.8
	MG	27.62	58.24	85.87	-	-	342.2	-	74.0	165.9	95.6	677.7
	BR	26.35	51.85	78.19	239.0	-	152.1	92.7	66.8	127.6	69.3	747.4
	BR+5	31.30	47.94	79.24	894.3	-	145.4	148.4	85.3	204.6	76.4	1,554.5
	BR+10	24.64	49.55	74.19	1,218.5	-	156.1	212.9	112.3	275.1	76.7	2,051.5
	FR	25.75	47.60	73.35	700.6	-	124.6	219.3	109.9	282.5	72.4	1,509.4

FS, fruit set, MG, mature green, BR, breaker stage, FR, full red color fruit. Units of chlorophyll and carotenoid contents are μg/ml and μg/g DW, respectively.

The carotenoid accumulation profile was analyzed to determine the color of immature green and mature red fruits (Figure 1 and Table 1). Total carotenoids were highest in mature red fruits of “AC2212” (2,051 μg/g DW), followed by mature red fruits of “3501” (1,274 μg/g DW), “Long Sweet” (521 μg/g DW), and “3509” (389 μg/g DW) (Table 1). The major carotenoids were capsanthin and zeaxanthin. Capsanthin content was highest in “AC2212” (1,218 μg/g DW) and “3501” (795 μg/g DW), “3509” contained 305 μg/g DW, and “Long Sweet” contained 185 μg/g DW. Capsanthin was detected at the breaker stage in “AC2212,” “3501,” and “3509” and 5 days after the breaker stage (BR+5) in “Long Sweet.” Capsanthin content increased as the fruit matured. Similar to capsanthin, zeaxanthin content increased after the breaker stage during ripening. “3501” had the highest contents, 338 μg/g DW. The mature red fruit of “AC2212,” “Long Sweet,” and “3509” contained 219, 98, and 48 μg/g DW, respectively. The total carotenoid content of “AC2212” (2,051 μg/g DW) was higher than that of the other three accessions, as were the lutein, β-carotene, and β-cryptoxanthin contents. At the fully ripened red stage, the total carotenoid content of “Long Sweet” was greater than that of “3509,” but the sum of capsanthin and zeaxanthin content of “3509” (353 μg/g DW) was greater than that of “Long Sweet” (283 μg/g DW). β-Carotene was detected in three accessions at concentrations ranging from 14 to 138 g/g DW, regardless of developmental stage, with the exception of “AC2212.” β-Carotene was detected in all stages of “AC2212” (128–283 μg/g DW). α-Carotene levels were highest in “AC2212” (69–100 μg/g DW), and a trace amount of α-carotene was detected in mature green fruit from “3501” (8 μg/g DW). Lutein content decreased during fruit development and ripening in the three accessions. Capsanthin and zeaxanthin contents increased after the breaker stage, as did the total carotenoid contents. The correlation coefficients between total carotenoids and capsanthin and zeaxanthin contents were 0.89 and 0.73, respectively (Supplementary Figure 2). In addition, total carotenoid levels correlated positively with total chlorophyll and chlorophyll *b* contents (r = 0.57 and 0.64). We hypothesized a correlation between carotenoids and chlorophyll synthesis.

### The structural transformation from chloroplast to chromoplast in “3501” and “3509”

**FIGURE 2:**
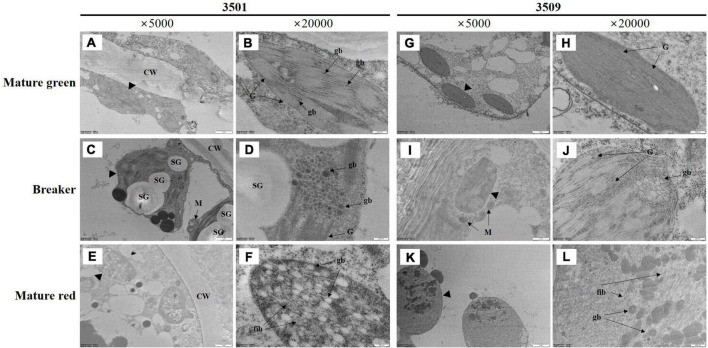
Transmission electron microscopy (TEM) image of plastids in mature green **(A,B,G,H)**, breaker stage **(C,D,I,J)**, and mature red **(E,F,K,L)** fruit in “3501” and “3509.” The second and fourth columns are enlarged images of the first and third columns. CW, cell wall, G, granum, gb, plastoglobuli, SG, starch granule, M, mitochondria, fib, fibrils.

To investigate changes in chromoplast structure accumulating carotenoids during fruit ripening, we conducted TEM image analysis to compare chloroplast and chromoplast structures of “3501” and “3509” (Figure 2). Although both “3501” and “3509” had green immature fruit and red mature fruit, the chlorophyll and carotenoid contents of the two accessions were distinct (Table 1 and Figure 1). There were no variations in chloroplast structure between “3501” and “3509” at the immature green stage (Figures 2A,B,G,H), although the darkness of green fruit and the content of total chlorophyll were discernible (Figure 1 and Table 1). A lot of grana, stacked structure of thylakoids, were observed at this stage. Plastoglobuli were small and sparsely distributed within grana in the chloroplast of immature green fruit. Except for starch granules, the transition state of the chloroplast to chromoplast at the breaker stage had a similar shape between “3501” and “3509” (Figures 2C,D,I,J). The plastid of the breaker stage fruit included grana and plastoglobuli. In the breaker stage fruit, many plastoglobuli were grouped and one to three clusters started to appear. The chromoplasts in mature red fruit from “3501” were different from those in mature red fruit from “3509” (Figures 2E,F,K,L), as the darkness of red fruit, and the total carotenoids content were distinguished (Figure 1 and Table 1). Plastoglobuli were dispersed throughout the chromoplast of “3501,” and fibril-containing plastoglobuli were jumbled together. Unlike “3501,” mixed plastoglobuli and fibril structures appeared on one side only in “3509.”

### Distribution of chlorophyll and carotenoid contents in recombinant inbred line populations

Two RIL populations, LA F_7:9_ RILs and FC RILs, were used to investigate the genetic factors regulating the green color intensity and carotenoid content. The green color intensity of the fruit was evaluated by visual assessment in LA RILs. The light green immature fruit color of “Long Sweet” was set as class 1 and the dark green color of “AC2212” as class 5. A total of 27, 84, 75, 23, and 44 RILs were classified as class 1, 2, 3, 4, and 5 in green color intensity, respectively, suggesting that the intensity of green color may be controlled by multiple genes (Supplementary Figure 3C).

**FIGURE 3:**
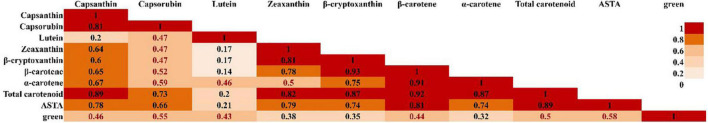
Pearson correlation heatmap between carotenoid color pigments of fruit in LA RILs. In the figure, red color indicates a strong correlation coefficient and light orange indicates a weak correlation coefficient.

The content distribution of carotenoids in LA and FC RIL populations showed skewed distribution, especially in total carotenoid content, capsanthin, and capsorubin. Capsanthin, capsorubin, and β-carotene contents were analyzed in the FC RIL population (Supplementary Figures 3, 5). Total carotenoids were calculated by the sum of the three pigments. The total carotenoid contents of FC RILs ranged from 1,695 to 9,234 μg/g DW, while that of LA RILs ranged from 420 to 5,531 μg/g DW. The distribution of two RILs exhibited continuous variation. Capsanthin and β-carotene were most abundant in FC RILs (Supplementary Figures 5A,C). Capsanthin and β-carotene contents ranged from 0 to 4,349 and 1,068 to 6,756 μg/g DW, respectively, in this population. β-Carotene content was higher than capsanthin content. Capsorubin contents ranged from 0 to 452 μg/g DW (Supplementary Figure 5B). Capsanthin and β-carotene, among seven carotenoids, were the two major pigments in the LA RIL population. Capsanthin content ranged from 119 to 2,302 μg/g DW, while β-carotene ranged from 40 to 3,097 μg/g DW (Supplementary Figures 3D,H). Lutein (4–94 μg/g DW) and α-carotene (20–301 μg/g DW) were not detected in some RILs (72 and 53) (data not shown). When the correlation between carotenoid pigments was analyzed, the total carotenoid content showed a high correlation with most carotenoid content, except capsorubin and lutein (Figure 3).

The American spice trade association color unit value analysis was also used for the measurement of total carotenoid content. The ASTA values ranged from 58 to 382 in LA RILs (Supplementary Figure 3B), whereas, ASTA values of FC F_5:7_ RILs were from 48 to 184 and F_7:9_ RILs were from11.54 to 136.17 (Supplementary Figure 5E). The ASTA value distribution also showed continuous variation like carotenoid content measured by HPLC, even though those of F_5:7_ RILs shifted to lower values. ASTA value showed a high correlation with total carotenoid and β-carotene contents, but the correlation coefficient was not as high as the correlation between total carotenoid and other pigments. Lutein contents had no significant correlation with the rest of the carotenoid contents, but a low correlation with α-carotene and green intensity. The green color intensity and ASTA values showed similar patterns (Supplementary Figures 3B,C).

### Gene expression related to carotenoid and chlorophyll biosynthesis during fruit ripening

**FIGURE 4:**
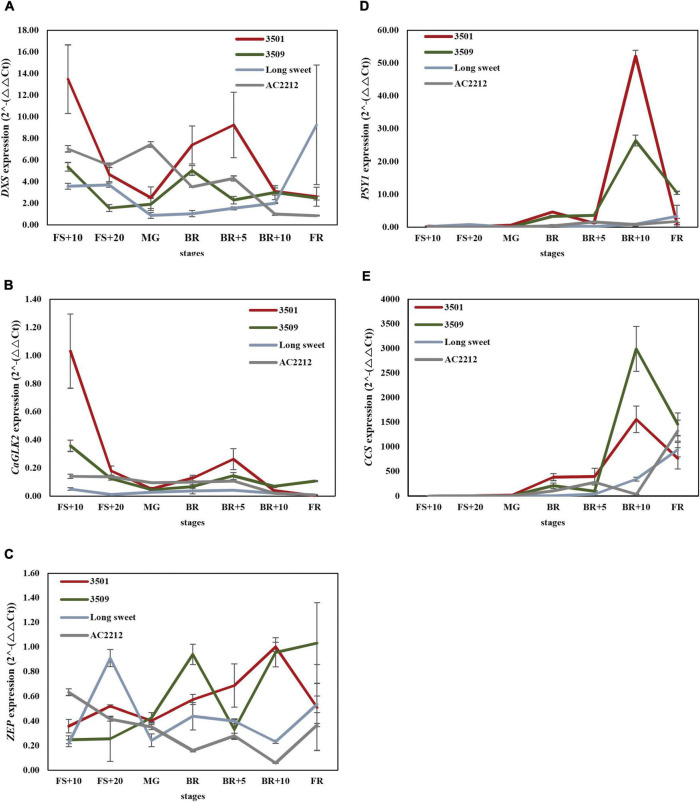
Expression analysis of **(A)** *DXS*, **(B)** *CaGLK2*, **(C)** *ZEP*, **(D)** *PSY1*, and **(E)** *CCS*. FS, fruit set, MG, mature green, BK, breaker stage, FR, full red color fruit. Red, green, light blue, and gray lines indicate expression patterns of “3501,” “3509,” “Long Sweet,” and “AC2212,” respectively.

To reveal the expression profiles of major genes during fruit ripening, we analyzed the expression of four genes during fruit development and ripening: *1-deoxy-d-xylulose 5-phosphate synthase* (*DXS*) involved in methylerythritol 4-phosphate (MEP) pathway for chlorophyll and carotenoid synthesis, *Capsicum annuum GOLDEN2-like* (*CaGLK2*) transcription factor in regulating chloroplast development in pepper fruit, *Phytoene synthase 1* (*PSY1*) encoding the first committed enzyme in carotenogenesis, and *Capsanthin-capsorubin synthase* (*CCS*), which esterifies xanthophylls to generate unique pigments of red pepper (Figure 4).

Significant differences were observed during fruit ripening in *DXS*, *CaGLK2*, *CCS*, and *PSY* expression levels between accessions. *DXS* expression levels of “3501” and “3509” were the highest at the FS+10 stage and increased again after the BR stage (Figure 4A). However, the *DXS* expression of “AC2212” was significantly higher in the green color stages compared to the breaker and mature stages. *DXS* expression of “Long Sweet” was lower than the three accessions at the FS+10 and MG to BR+5 stages but was highest at the FR stage. The expression levels of *CaGLK2* were high at the early stage (FS+10) and eventually reduced in “3501” and “3509” (Figure 4B). During the fruit ripening, the expression level of *CaGLK2* in “Long Sweet” did not show significant changes. *CaGLK2* expression level in “AC2212” gradually decreased. Four accessions showed similar low expression levels after the BR+10 stage.

In contrast to two genes, *DXS* and *CaGLK2*, three genes, *PSY1, ZEP*, and *CCS*, which regulate carotenoid biosynthesis, showed distinct expression patterns (Figures 4C–E). In the four accessions, the expression levels of the two genes started to increase after the BR stage. The expression levels of these genes peaked at BR+10 in “3501” and “3509,” whereas, “AC2212” and “Long Sweet” showed distinct patterns. *PSY1* of “3501” and “3509” showed no significant differences in expression during ripening (Figure 4D). At BR+10, “3501” and “3509” showed the highest levels of expression, but “AC2212” and “Long Sweet” did not show significant changes during ripening. *ZEP* expression increased during ripening and peaked at the BR+10 stage in “3501” (Figure 4C). *ZEP* expression of “3509” peaked at BR and then increased again in BR+10. ZEP was highly expressed in “Long Sweet” at FS+20. On the other hand, *ZEP* expression decreased gradually during ripening in “AC2212.” Similar to *PSY1*, after the BR stage, *CCS* of “3501” and “3509” increased expression dramatically at BR+10 (Figure 4E). “3509” had the highest level than “3501” at BR+5, while “AC2212” had the lowest level at BR+10. At FR, “AC2212” and “Long Sweet” had the highest level of other stages. We compared the metabolite profiles to patterns of gene expression (Supplementary Figure 2). There was a significant positive correlation between zeaxanthin concentration and *CCS* and *PSY1* expression (*r* = 0.51 and 0.58). In addition, gene expression patterns were compared. *CCS* expression was significantly correlated with *PSY1* and *ZEP* expression (r = 0.70 and 0.58, respectively), and *PSY1* expression was similarly correlated with *ZEP* expression (r = 0.59). Additionally, there was a significant correlation between *DXS* expression and *CaGLK2* expression (r = 0.70). These findings suggest that *CaGLK2* is associated with carotenoid synthesis regulation.

### Correlation between the expression of chlorophyll-related genes and green color intensity

**FIGURE 5:**
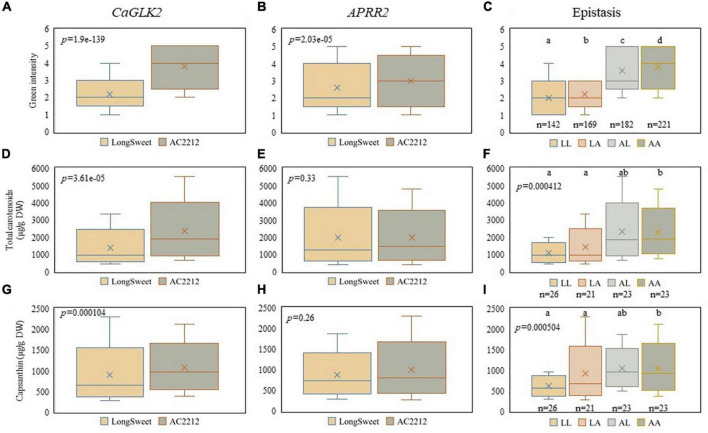
Comparison between genes related to chlorophyll synthesis and carotenoid contents. Boxplots of green fruit color intensity distribution and **(A)** *CaGLK2*, **(B)** *APRR2*, and **(C)** both genes. Boxplots of total carotenoid contents (μg/g DW) distribution and **(D)** *CaGLK2*, **(E)** *APRR2*, and **(F)** both genes. Capsanthin contents (μg/g DW) distribution regulated by **(G)** *CaGLK2*, **(H)** *APRR2*, and **(I)** interaction of two genes.

Since the upstream steps of the pathway of chlorophyll biosynthesis also form the upstream pathway of carotenoid biosynthesis, we examined the correlation between chlorophyll biosynthesis genes and carotenoid content. Green fruit color intensity and carotenoid content in LA RILs were compared to *CaGLK2* and *APRR2* genotypes (Figure 5). Plants with the “Long Sweet” and “AC2212” genotypes had significantly different green fruit color distributions (Figures 5A–C). When plants with “AC2212” genotypes of *CaGLK2* and *APRR2* were compared, the green fruit color of “AC2212” genotype plants were considerably darker (Figure 5C). When RILs had the “AC2212” genotype in *CaGLK2*, the total carotenoid content distribution was significantly higher (Figure 5D). In *APRR2*, however, no variations in total carotenoid concentration were found between the two plant groups (Figure 5E). Only when the *CaGLK2* genotype was altered, the statistical analysis of two gene interactions demonstrated a significant difference (Figure 5F). The distribution of capsanthin content in each of the two genotype groups increased in parallel to the total carotenoid content (Figures 5G–I). *CaGLK2* expression was highly correlated with the carotenoid content of LA RILs. Taken together with the correlation between *CaGLK2* and *DXS* expression in parental lines, carotenoid content may be associated with chlorophyll biogenesis.

### Quantitative trait locus analysis of carotenoids and chlorophylls contents using recombinant inbred line populations

To identify QTLs for carotenoid and chlorophyll contents, linkage maps were generated using GBS. “LA” genetic map was composed of 1,093 SNPs polymorphic between “Long Sweet” and “AC2212” (Supplementary Tables 2, 3). A total of 1,093 SNPs were divided into 13 linkage groups corresponding to the “Dempsey” genome reference except chromosome 8. The total length of the “LA” map was 1432.8 cM with the average length of 13 linkage groups of 110.22 cM, and the length between SNPs was approximately 1.31 cM. The average number of SNPs per linkage group was 84.08. The “FC” genetic map was composed of 539 SNPs (Supplementary Table 3). A total of 12 linkage groups of FC RILs also corresponded to the “Dempsey” genome. The total length of the “FC” map was 1972 cM. Except for a-carotene and lutein, the distributions of seven carotenoid contents of LA RILs were mostly skewed (Supplementary Figures 3, 4 and Supplementary Table 4). The distributions of ASTA value and total carotenoid content were also skewed. Carotenoid contents of FC RILs, excluding capsorubin, showed similarly skewed distributions (Supplementary Figures 5, 6 and Supplementary Table 4).

**FIGURE 6:**
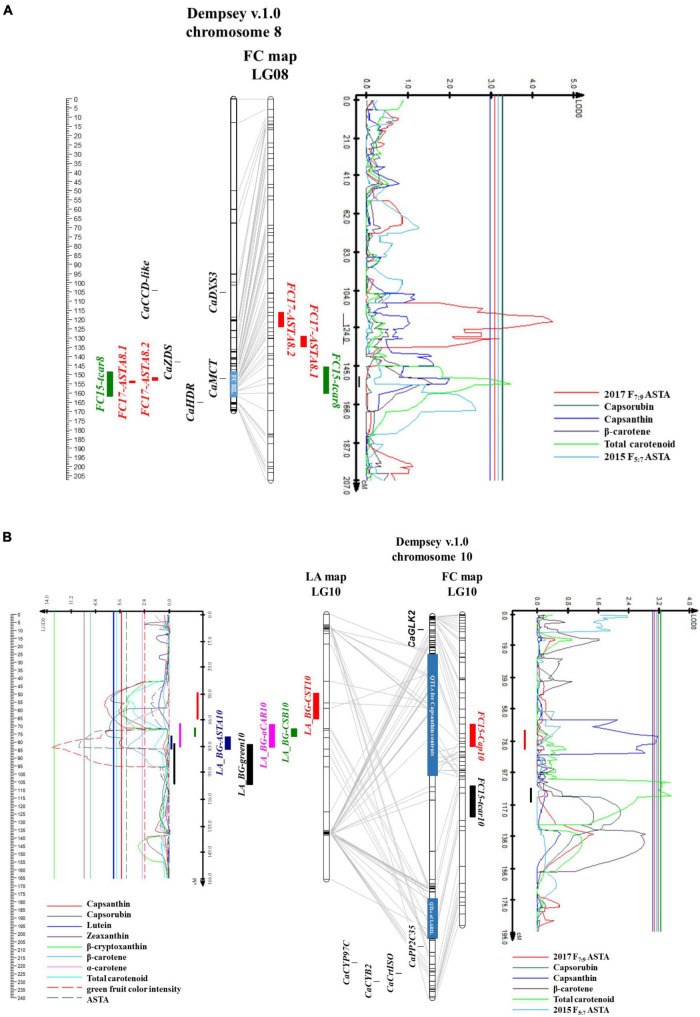
Physical map based on Dempsey genome, linkage maps of RILs, and QTL likelihood profile containing the QTL positions. **(A)** Chromosome 8 and linkage group 8 of FC map, **(B)** chromosome 10 and linkage group 10 of LA and FC map.

Three QTLs for carotenoid content were detected in LA RILs. Three QTLs were detected for capsanthin, capsorubin, and ASTA value, respectively (Table 2). The QTLs were mostly located on chromosome 10. LOD ranged from 7.39 to 13.59, and the phenotypic variation explained by the QTLs ranged from 19.8 to 43.2%. QTLs for capsanthin, capsorubin, and ASTA value were detected on chromosome 10 (Figure 6B). By contrast, no QTLs were detected on lutein, zeaxanthin, β-cryptoxanthin, and total carotenoid contents.

**TABLE 2:** Quantitative trait loci (QTLs) for carotenoid contents and green intensity of LA recombinant inbred lines (RILs).

Pop.	Trait	QTL	Chr.	Marker	LOD	R^2^ (%)	Location (cM)	Location (Mbp)	Additive effect
LA RILs	Capsanthin	*LA_BG-CST10*	10	LA10_18	7.4	20.5	49.4–65.7	37.5–58.3	187.59^a^
	Capsorubin	*LA_BG-CSB10*	10	LA10_22	7.29	19.8	71.6–76.6	178.2–182.1	14.71^a^
	a-carotene	*LA_BG-aCAR1*	1	LA1_1	4.35	19.4	0–3.3	10.1–11.2	34.73^a^
		*LA_BG-aCAR10*	10	LA10_24	5.78	31.5	68.8–83.3	37.5–192.6	42.71^a^
	Green fruit color	*LA_BG-green10*	10	LA10_24	13.56	43.2	81.4–106.7	192.6–204	0.97^a^
	ASTA	*LA_BG-ASTA10*	10	LA10_24	10.36	27.4	76.6–84.6	182.1–203.2	33.11^a^
FC RILs	ASTA (F7:9)	*FC17-ASTA4*	Chr.4	FC4_43	3.1	6	172.7–176.9	0.483–0.485	−5.92^b^
		*FC17-ASTA8.1*	Chr.8	FC8_22	3.3	6.6	72.5–78.4	156.5–157.7	6.18^b^
		*FC17-ASTA8.2*	Chr.8	FC8_27	4.5	8.9	83.4–91.5	154.8–156.3	7.20^b^
	Capsanthin	*FC15-Cap10*	Chr.10	FC10_38	3.2	7.4	68.6–82.7	24.4–100.4	0.25^b^
	Total carotenoids	*FC15-tcar8*	Chr.8	FC8_15	3.5	7.7	47–61.8	151.6–165.0	0.42^b^
		*FC15-tcar10*	Chr.10	FC10_60	3.5	8.1	107.2–126.9	148.3–171.7	0.43^b^

Additive effect: Positive additivity indicates that the QTL alleles from “AC2212” and “3501” in LA and FC RILs, respectively, originating increase the phenotype values, whereas negative additivity indicates that the QTL alleles originating from “Long Sweet” and “3509” in LA and FC RILs, respectively increase the phenotype values. ^a^μg/g DW is the unit of an additive effect in LA RILs. ^b^μg/g DW is the unit of an additive effect in FC RILs.

The upper arm of chromosome 10 contained QTLs, *LG_BG-CST10* for capsanthin contents (Figure 6B). These were located between 37.5 and 58.3 Mbp of the Dempsey reference genome. In the QTL regions, no genes regulating carotenoid biosynthesis were found, while *CaGLK2* was located 10.78 Mbp away from the QTL. *LA_BG-CSB10*, QTL for capsorubin content, was located on the lower arm of chromosome 10, 178.2–182.1 Mbp, and QTL, *LA_BG-ASTA10*, for ASTA value, was located on the flanking region of *LA_BG-CSB10* between 182.1 and 203.2 Mbp. *LA_BG-CST10*, *LA_BG-CSB10*, and *LA_BG-ASTA10* overlapped with the *LA_BG-αCAR10* region. On chromosome 10 of the LA genetic map, *LA_BG-CSB10* and *LA_BG-ASTA10* were located 71.6–84.6 cM side by side. However, *LA_BG-CST10* was located separately. *CaPP2C35* was at 207.9 Mbp by flanking with *LA_BG-green10* (Wu et al., 2022). Furthermore, carotenoid biosynthesis genes, *CaCrtISO*, *CaLCYB2*, and *CaCYP97C* were clustered on 218.13–229.91 Mpb. In LA RILs, all QTLs for capsanthin, capsorubin, ASTA value, and green fruit color intensity exhibited additive effects. In other words, these two metabolite contents and the ASTA value were higher in plants with the “AC2212” genotypes for QTLs in LA RILs (Table 2).

Six QTLs related to carotenoid content were detected in FC RILs (Table 2). One QTL for capsanthin was detected on chromosome 10, and two QTLs for total carotenoid content were detected on chromosome 8 and 10, respectively. Additionally, three QTLs for ASTA value of F_7:9_ RILs were located on chromosomes 4 and 8. LOD ranged from 3.1 to 4.5, and the phenotypic variation explained by the QTLs was 6–8.9%. No QTL was detected for capsorubin and β-carotene. Two QTLs, *FC15-Cap10*, for capsanthin content and *FC15-tcar10* for total carotenoid contents were located at 24.4–100.4 Mbp and 148.3-171.7 Mbp on chromosome 10 (Figure 6B). The regions of these two QTLs covered the *LA_BG-CST10* region. *FC15-tcar8* for carotenoid content was located at 151.6-165 Mbp of chromosome 8 (Figure 6A). Two QTLs for ASTA value, *FC17-ASTA8.2* and *FC17-ASTA8.1*, were located between 154.8 and 157.7 Mbp side by side, and these overlapped on *FC15-tcar8*. However, these three QTLs were located in independent linkage groups. Nevertheless, MEP cytidylyltransferase 2 (*CaMCT2*) involved in the MEP pathway was located within the QTLs region. In addition, one QTL related to ASTA value was between 483 and 485 kbp, the upper arm of chromosome 4 (Supplementary Figure 7B). In FC RILs, most QTLs for ASTA value, capsanthin, and total carotenoid contents had an additive effect (Table 2). However, only QTLs for the ASTA value in chromosome 4 (*FC17-ASTA4*) showed a negative additive effect. FC RILs with the “3509” genotype for *FC17-ASTA4* had a lower ASTA value, whereas FC RILs with the “3501” genotype in other QTLs on chromosomes 4, 8, and 10 had a higher ASTA value, capsanthin, and total carotenoid contents.

One QTL for green fruit color intensity of LA RILs was detected on chromosome 10 like other QTLs for carotenoid contents (Figure 6B). The region of this QTL was between 192.6 and 204 Mbp, which overlapped with the region of *LA_BG-ASTA10*.

## Discussion

Genetic research on carotenoid biosynthesis has been subjected to the role of structural genes in carotenogenesis. Only limited studies on the quantitative genetic factors for carotenoid content in pepper have been reported, even though carotenoid content in fruit is a quantitative trait, and breeders and consumers need high-pigmented red varieties. In this study, the genetic correlation between chlorophylls and carotenoid synthesis was investigated by gene expression analysis and QTL analysis for carotenoid content and green immature fruit color intensity.

Similar chlorophyll content patterns were observed in “3501” and “3509” during ripening. Chlorophyll contents were decreased in “3501” and “3509” during fruit color break and ripening (Table 1). In other words, this result could be interpreted that the chlorophyll synthesis was reduced during plastid conversion. By contrast, the chlorophyll contents of “Long Sweet” and “AC2212” after the breaker stage were not decreased as much as “3501” and “3509.” Among four accessions, “Long Sweet” possessed the lowest chlorophyll content before BR+10. Even though “Long Sweet” contained more total carotenoids than “3509,” the contents of its red pigment, capsanthin, and capsorubin, were lower than those of the other three accessions. Thus, mature “Long Sweet” fruit had a light red color. The pattern of chlorophyll content during fruit ripening in “Long Sweet” correlated with the expression pattern of *DXS* and *CaGLK2* (Figure 4). The red color development of fruit might be caused not only by carotenoid synthesis but also by a reduction of chlorophyll synthesis. Among four accessions, “AC2212” had the highest levels of chlorophyll and carotenoids. chlorophyll levels of “AC2212” did not decrease significantly during ripening. Therefore, mature “AC2212” fruits appeared dark brown. *DXS* expression until green maturity was higher than those in “AC2212” than in “3509” and “Long Sweet,” which had light green fruits. *CaGLK2* expression was not higher than “3501” and “3509.” However, the chlorophyll content of “AC2212” was even higher than that of “3501.” To investigate the correlation between chlorophyll and carotenoid contents in brown fruit, it is necessary to examine additional genes for chloroplast development and degradation. The mature brown color of “AC2212” would be affected by the *CaSGR* gene, which inhibits the degradation of chlorophyll in fruit (Borovsky and Paran, 2008).

Carotenoid synthesis and storage in pepper fruit occur in spindle-shaped fibrils in chromoplast (Sun et al., 2018). TEM image analysis of “3501” and “3509” showed significant differences in chromoplast structure as well as carotenoid concentration. Chromoplast of “3501” mature red fruit had a denser fibril structure containing a lot of plastoglobuli (Figure 2). This result agrees with the previous observation by Berry et al. (2019).

Multiple genes controlling carotenoid biosynthesis flux have been reported by overexpression or silencing studies. *DXS* and *DXR* encode rate-limiting enzymes for MEP biosynthesis. Overexpression of *DXS* in Arabidopsis seedlings increased total carotenoid contents (Nisar et al., 2015). Similar to *DXS*, *PSY* is also considered a rate-determining step gene regulating phytoene synthesis, a precursor for carotenes (Nisar et al., 2015). *DXS* and *PSY* expressions in pepper were similar (Berry et al., 2019). Capsanthin content is regulated by *DXS* and *PSY* expression. Figures 4A,D show corresponding results to previous reports. *DXS* and *PSY1* expression of “3501” containing a high level of capsanthin, and total carotenoid were higher than “3509” and “Long Sweet” before being fully mature red. The expression level of *DXS* and *PSY1* in “3501” was the highest, nevertheless, the expression patterns of the two genes were different. *PSY1* expression started at the breaker stage when the fruit development finished, in contrast, *DXS* expression starts at the initial development stage. Even though “AC2212” had the highest chlorophyll and carotenoid content, the *PSY1* expression level was similar to “Long Sweet.” In addition, the expression level of “AC2212” *DXS* was lower than “3501” in most stages and gradually decreased. Consequently, it is assumed that another genetic factor in the upstream step or transcription affects the intensity of the red color. *CCS* expression pattern was comparable to the *PSY1* expression pattern. After the breaker stages, however, the *CCS* expression level in “3509” was higher than in other accessions. In addition, “AC2212” had lower *CCS* expression levels than “3501” and “3509,” except the fully red stage. This situation differed from the accumulation of carotenoids in four accessions and corresponds with prior research suggesting that *CCS* had no significant correlation with color intensity (Berry et al., 2019).

Very limited studies were reported on the QTL analysis for capsanthin and total carotenoid contents in pepper. QTLs for capsanthin contents, *Cst15.1* and *Cst13.1*, were reported recently (Konishi et al., 2019). *Cst15.1* on chromosome 9 was generated from not fully mature red fruit (45 DAF), and *Cst13.1* on chromosome 6 was detected by fully mature red fruit (90 DAF). Major QTLs of LA RILs and FC RILs were detected on chromosomes 8 and 10, respectively. *LA_BG-CST10* for capsanthin contents in LA RILs located between 24.4 and 100.4 Mbp of chromosome 10, and *LA_BG-ASTA10*, QTLs for ASTA color value in LA RILs were between 182.1 and 203.2 Mbp of chromosome 10 (Figure 6B). *LA_BG-αCAR1*, another QTL associated with α-carotene contents was detected between 10.1 and 11.2 Mbp on chromosome 1 (Supplementary Figure 7A). On the upper arm of chromosome 1, transcription factors regulating chloroplast development, *CcLOL1*, *APRR2*, and *SGR* were clustered between 8.37 and 19.89 Mpb. In addition, *CaHMGS1* and *CaMDS*, involving MVA and MEP pathway were at 8.19 and 3.89 Mbp, respectively. *FC15-tcar8* and *FC17-ASTA8.1* and *FC17-ASTA8.2* for total carotenoid contents and ASTA value in FC RILs were between 151.6 and 165 Mbp of chromosome 8 (Figure 6A). To delimit the QTLs region for capsanthin contents, LA and FC genetic maps have to be modified to high resolution because this region was large and recombinations existed between SNPs. Despite the large QTL region, no candidate genes were found regulating carotenoid metabolism. Transcriptome analysis is necessary to find candidate genes in this region.

We demonstrated the correlation between carotenoid biosynthesis flux and chlorophyll synthesis gene expression. It was reported that *DXS* was expressed at the stage of fruit color change (Berry et al., 2019); however, the expression level of *DXS* was also higher at the initiation stage of fruit development (Figure 4A). The *DXS* expression pattern at the early stage of fruit ripening was similar to the *CaGLK2* expression. Carotenoid contents of LA RILs changed according to the *CaGLK2* genotype (Figure 5). In addition, QTL regions for ASTA value, green immature fruit color intensity, capsanthin, and α-carotene contents of the LA genetic map were clustered on chromosome 10 (Figure 6B). Thus, it is assumed that candidate genes might be related to GGPP synthesis, which is a common precursor of chlorophylls and carotenoid biosynthesis. QTLs for LA RILs on chromosome 10 were far from *CaGLK2* but several genes involved in the carotenoid metabolism pathway are in the lower arm, i.e., *CaCYP97C* for α-carotene hydroxylase at 218.1 Mbp; *CaLCYB2*, lycopene β-cyclase2 at 229.9 Mbp; and *CaCrtISO* for phytoene isomerase to produce lycopene. These three genes are involved in the upstream regulation of carotenogenesis before xanthophyll synthesis. According to Guo et al. (2020), these genes express low to medium levels in the pericarp of mature green fruit and decrease gradually during ripening. The region of QTLs for total carotenoid content and ASTA value in FC RILs contained *CaMCT2* involved in the MEP pathway and located in the chloroplast (Figure 6B).

Total carotenoid contents in red pepper fruit had a strong correlation with the contents of red pigmented xanthophylls (Figure 3). This phenomenon is consistent with previous studies (Giuffrida et al., 2011; Jeong et al., 2019). Since orange a-carotene and yellow lutein were separately synthesized by lycopene 3-cyclase (LycE) and/or lycopene b-cyclase (LycB), these two carotenoids correlated less with other carotenoid contents including capsanthin. ASTA color value was considered as the representative trait of total carotenoid contents for QTL mapping. This method is used to examine paprika quality in western countries and the high pigment in chili pepper in Asian countries. When ASTA value in LA RILs was compared to each carotenoid content, a strong correlation between ASTA value and β-carotene, the downstream pigments contents, and total carotenoid content was confirmed, even though α-carotene and lutein were not correlated with ASTA value (Figure 3). Yuan et al. (2015) showed similar results in 523 *C. annuum* accessions. ASTA value showed a strong correlation with xanthophylls and total carotenoid content excluding lutein, α-carotene, and β-carotene. QTL for ASTA value in LA RILs was overlapped on QTLs for other carotenoid contents, and QTLs for ASTA value in FC RILs were overlapped on QTL for total carotenoid content. This implies that the ASTA value unit can be the alternative trait for total carotenoid content and be a breeding tool for the development of high pigment pepper varieties.

Chlorophyll is composed of a chlorin ring called chlorophyllide and phytyl side chain, isoprenoid alcohol formed by 20 carbons (Gutbrod et al., 2019). Geranylgeranyl diphosphate (GGPP) is reduced and converted to phytyl moiety of chlorophyll *a* in the thylakoid membrane, and light-harvesting tissues show a green color phenotype. During senescence, the number of thylakoid membranes is decreased, chlorophylls are degraded, and chlorophyll *a* is decomposed into phytol and pheophorbide a. Therefore, the synthesis of the phytyl side chain from GGPP might regulate chlorophyll contents. If GGPP synthase, GGPPS involving isoprenoids pathway, have mutations, a variegated phenotype has appeared, and chlorophylls and carotenoids contents were decreased (Ruiz−Sola et al., 2016). In this study, QTLs for immature fruit color intensity and carotenoid contents were clustered and QTLs for capsanthin contents were overlapped on chromosome 10. QTLs for total carotenoid contents were detected on chromosome 8. This QTLs region contains *CaMCT2* involved in chlorophyll and carotenoid precursor biosynthesis. Genetic factors for chlorophylls and carotenoid biosynthesis flux might be regulated by upstream genes of carotenoid metabolism or isoprenoid biosynthetic pathways.

In conclusion, we have shown that chlorophyll and carotenoid production are linked in *Capsicum*. *CaGLK2* for chloroplast development was correlated with both carotenoid and chlorophyll content in the RIL population, as determined by genotype analysis. Furthermore, QTLs on chromosomes 8 and 10 were associated with both chlorophyll and carotenoid biosynthesis. This study suggests a new direction for the investigation of qualitative and quantitative genetic factors regulating carotenoid content and breeding new varieties containing high levels of carotenoid content in crop plants.

## Data availability statement

The data presented in this study are deposited in the Figshare data repository at https://doi.org/10.6084/m9.figshare.20972488.v1.

## Author contributions

B-CK, SJ, KH, and YK conceived and designed the experiments. KH and YK constructed two RIL populations. SJ, KH, and YK analyzed the genotype and phenotype of two RIL populations. SJ and JJ analyzed the GBS data. GK conducted the statistical analysis. SJ and S-YL conducted HPLC and TEM microscopy. J-KK supervised TEM microscopy. B-CK and SJ drafted and revised the manuscript. B-CK supervised the overall processes. All authors contributed to the article and approved the submitted version.

## Supplementary material

Supplementary Figure 1 Green classification of green immature fruit on LA RILs. The green intensity was divided into five classes. Light green was class 1 referring to “Long Sweet” and Dark green was class 5 referring to “AC2212”.

Supplementary Figure 2 Correlation between carotenoid pigment contents and gene expression levels. In the figure, red colors and blue colors indicate positive and negative correlation, respectively. The correlation coefficient marked with an asterisk indicates that its *p-*value is less than 0.05.

Supplementary Figure 3 Carotenoid pigments distribution in LA RILs. **(A)** total carotenoid contents (μg/g DW), **(B)** ASTA value, **(C)** Green fruit color intensity of green mature fruit, **(D)** Capsanthin contents (μg/g DW), **(E)** Capsorubin contents (μg/g DW), **(F)** Zeaxanthin contents (μg/g DW), **(G)** β-cryptoxanthin contents (μg/g DW), **(H)** β-carotene contents (μg/g DW). **(A,D–H)** Carotenoid contents were measured in 96 RILs. **(B,C)** ASTA value and green fruit color intensity were measured in 254 RILs.

Supplementary Figure 4 Quantile-quantile plot to verify the distribution of carotenoid contents in LA RILs. Q-Q plots of **(A)** total carotenoid contents, **(B)** ASTA value, **(C)** capsanthin, **(D)** capsorubin, **(E)** zeaxanthin, **(F)** β-cryptoxanthin, and (G) β-carotene contents distribution. Red diagonal line in plots represents a theoretical normal distribution.

Supplementary Figure 5 Carotenoid pigments distribution in FC RILs. **(A)** Capsanthin contents, **(B)** Capsorubin contents, **(C)** β-carotene contents, and **(D)** total carotenoid contents (mg/g DW). **(A–D)** Carotenoid contents were measured in F_5:7_ generation. **(E)** ASTA value, ASTA values were analyzed in both F_5:7_ and F_7:9_ RILs.

Supplementary Figure 6 Quantile-quantile plot to verify the distribution of carotenoid contents in FC RILs. Q-Q plots of **(A)** capsanthin, **(B)** β-carotene, **(C)** total carotenoid contents, and **(D)** ASTA value distribution. Red diagonal line in plots represents a theoretical normal distribution.

Supplementary Figure 7 Physical map based on Dempsey genome, linkage maps of RILs, and QTL likelihood profile containing the QTL positions. **(A)** Chromosome 1 and linkage group 1 of LA map. Chromosome 1 is the left bar and linkage group 1 is the right bar. Genes for carotenoid and chlorophyll synthesis are indicated with black boxes and *LA_BG-aCAR1* with pink boxes. Graph for *LA_BG-aCAR1* is indicated with a pink line. **(B)** Chromosome 4 and linkage group 4 of FC map. Chromosome 4 is the left bar and linkage group 4 is the right bar. Genes for carotenoid and chlorophyll synthesis are indicated with black boxes and *FC17-ASTA4* with red boxes. Graph for *FC17-ASTA4* is indicated with a red line.
