# Supplementary material for: Investigation of genetic factors regulating chlorophyll and carotenoid biosynthesis in red pepper fruit
Source: Front Plant Sci. 2022 Sep 15;13:922963. doi: 10.3389/fpls.2022.922963 (PMC9521427; doi:10.3389/fpls.2022.922963)
Supplement: Supplementary file 1 [file Data_Sheet_1.docx]

**Supplementary Tables**

Supplementary Table S1. Primers for chlorophyll and carotenoid biosynthetic gene expression analysis.

| Primer name | Primer (5'-3') sequence | reference |
| --- | --- | --- |
| CaGLK2-F | GTGAAGGTTGATTGGACTCCAGAGC |  |
| CaGLK2-R | GTTTTCGATGAGCTCGATATTTTTG |  |
| PSY1-F | CGTTTTACTTAGGAACGATGCT |  |
| PSY1-R | CTGAAAACATCTTCTAGCCTGTCTTC |  |
| CCS-F | CCACATCAAAGCCAGAGTCTTTAG |  |
| CCS-R | GGTGAAGGGTCAACGCAACATACC |  |
| DXS-F | TACCAACCTCAAGGGGAATTCCTTTG |  |
| DXS-R | GGCTCCTTCTGATGAAGCGGAG |  |
| ZEP-F | GGTGGTGTGGATGCTCCAAA | Lee at al., 2021 |
| ZEP-R | ATTAGGCTGCATAGCATGGACT | Lee at al., 2021 |

Supplementary Table S2. Summary of LA genetic map.

| linkage group | # raw SNPs | # SNPs | size (cM) |
| --- | --- | --- | --- |
| LA01 | 3,715 | 113 | 163.5 |
| LA02 | 2,194 | 46 | 18.3 |
| LA03 | 3,250 | 90 | 111.3 |
| LA04 | 2,786 | 81 | 237.4 |
| LA05 | 2,783 | 84 | 114.7 |
| LA06 | 2,868 | 74 | 128.6 |
| LA07 | 3,056 | 55 | 97.7 |
| LA08-1 | 2,013 | 14 | 45.8 |
| LA08-2 |  | 21 | 48 |
| LA9 | 2,865 | 136 | 149.4 |
| LA10 | 2,745 | 83 | 165.8 |
| LA11 | 2,887 | 231 | 147.9 |
| LA12 | 2,795 | 65 | 4.4 |
| Total | 33,957 | 1093 | 1432.8 |

Supplementary Table S3. Summary of FC genetic map.

| linkage group | # raw SNPs | # SNPs | size (cM) |
| --- | --- | --- | --- |
| FC01 | 3,112 | 35 | 90.3 |
| FC02 | 2,203 | 18 | 66.6 |
| FC03 | 3,331 | 71 | 247.1 |
| FC04 | 1,931 | 44 | 178 |
| FC05 | 1,991 | 35 | 164.2 |
| FC06 | 2,533 | 56 | 204.2 |
| FC07 | 2,220 | 30 | 66.3 |
| FC08 | 2,073 | 72 | 207.3 |
| FC09 | 2,189 | 60 | 179.1 |
| FC10 | 2,077 | 61 | 194.6 |
| FC11 | 2,058 | 29 | 166.5 |
| FC12 | 2,217 | 36 | 183.9 |
| Total | 27,935 | 547 | 1,972.0 |

**Supplementary Table S4. Normality test of phenotype distribution in LA and FC RILs.**

| traits | LA RIL population | FC RIL population |
| --- | --- | --- |
| capsanthin | 0.2475 | 0.136 |
| capsorubin | 0.2141 | 5.43E-05 |
| lutein | 2.60E-14 |  |
| zeaxanthin | 0.005276 |  |
| β-cryptoxanthin | 0.0008059 |  |
| β-carotene | 0.008143 | 0.775 |
| α-carotene | 2.02E-08 |  |
| Total carotenoids | 0.1204 | 0.5418 |
| ASTA | 0.2781 | 0.7697 |

The values in the table indicate the *p-value* of the Kolmogorov-Smirnov test.
