# Supplementary figures and images for: Investigation of genetic factors regulating chlorophyll and carotenoid biosynthesis in red pepper fruit
Source: Front Plant Sci. 2022 Sep 15;13:922963. doi: 10.3389/fpls.2022.922963 (PMC9521427; doi:10.3389/fpls.2022.922963)

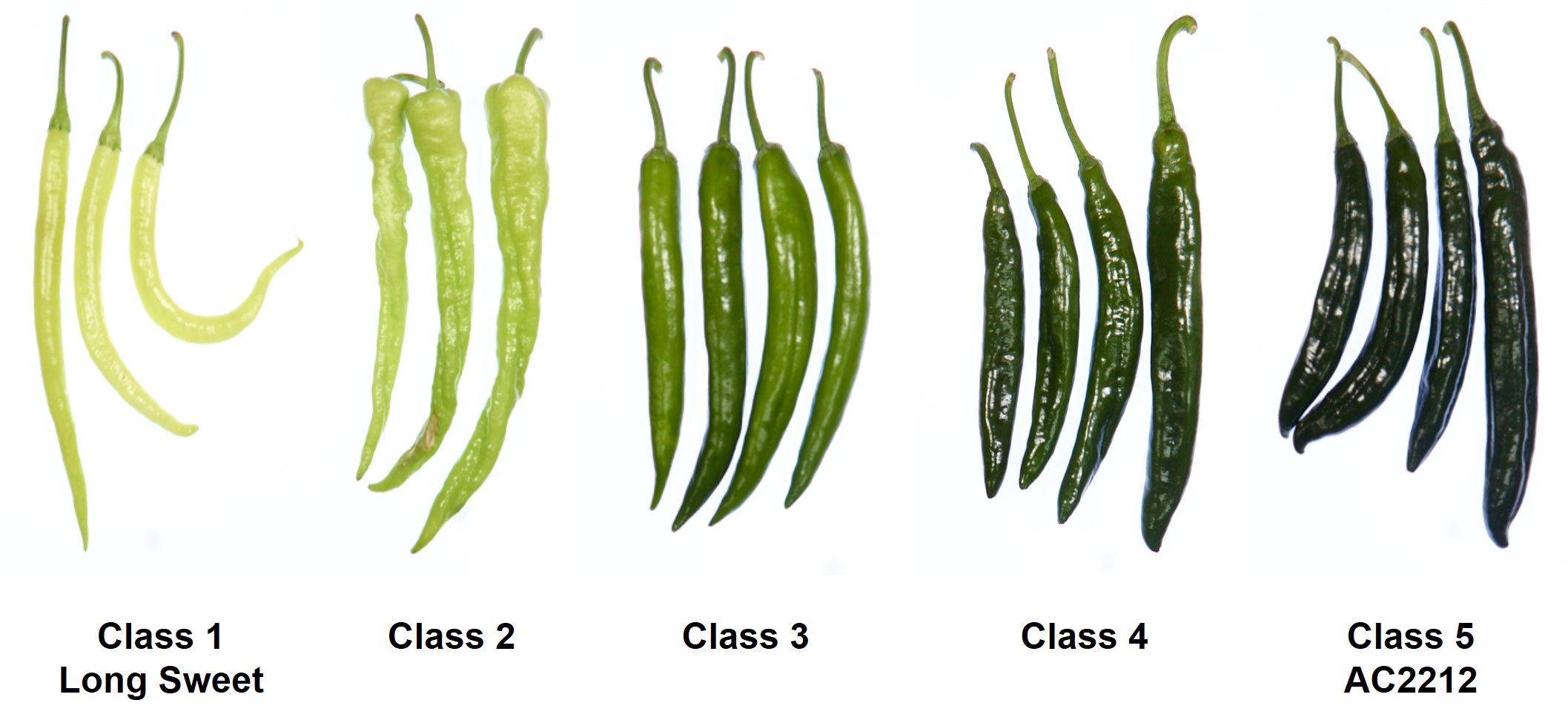

Supplement: Supplementary Figure 1 — Green classification of green immature fruit on LA RILs. The green intensity was divided into five classes. Light green was class 1 referring to “Long Sweet” and Dark green was class 5 referring to “AC2212”. [file Image_1.JPEG]

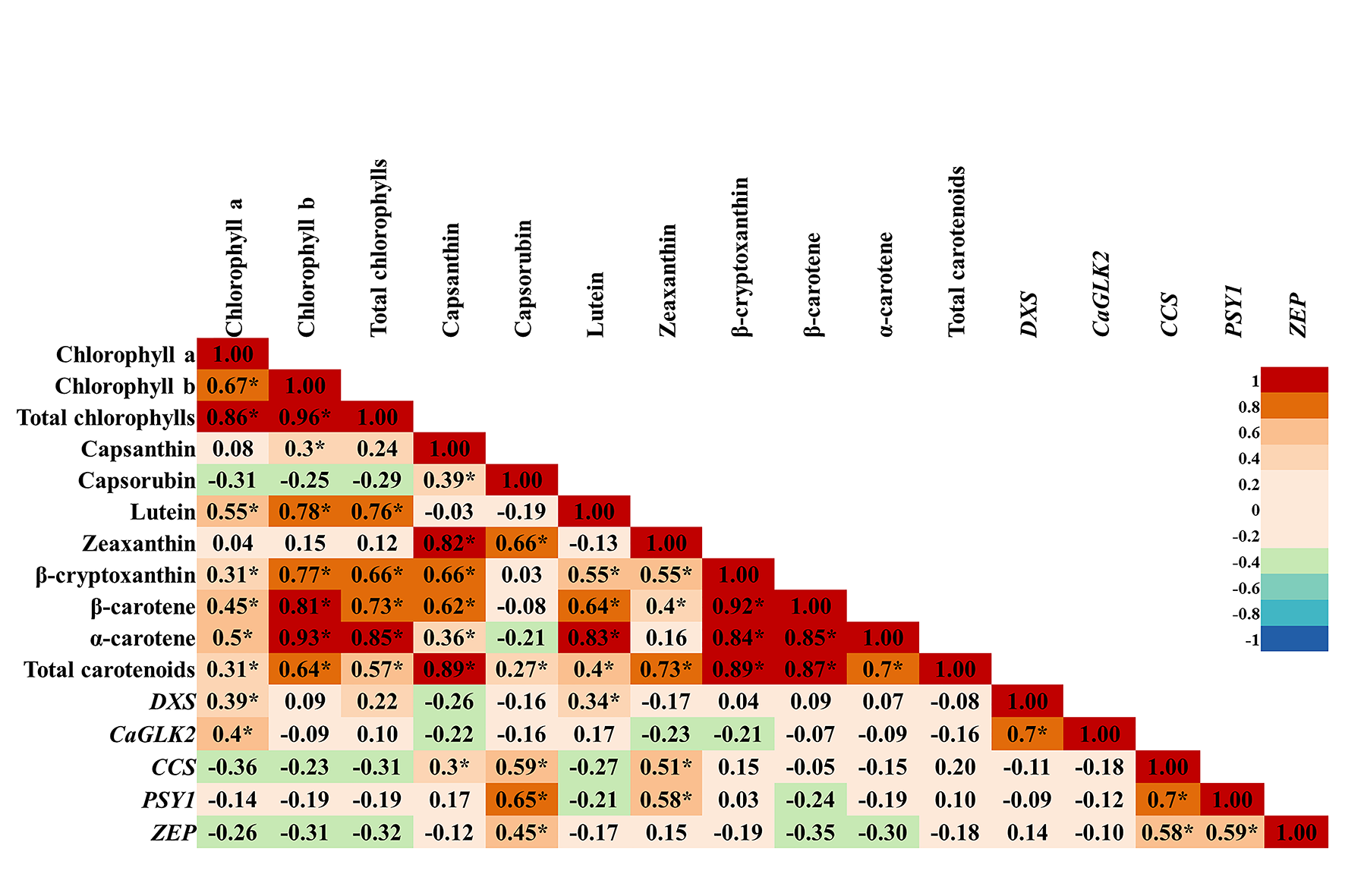

Supplement: Supplementary Figure 2 — Correlation between carotenoid pigment contents and gene expression levels. In the figure, red colors and blue colors indicate positive and negative correlation, respectively. The correlation coefficient marked with an asterisk indicates that its p-value is less than 0.05. [file Image_2.TIF]

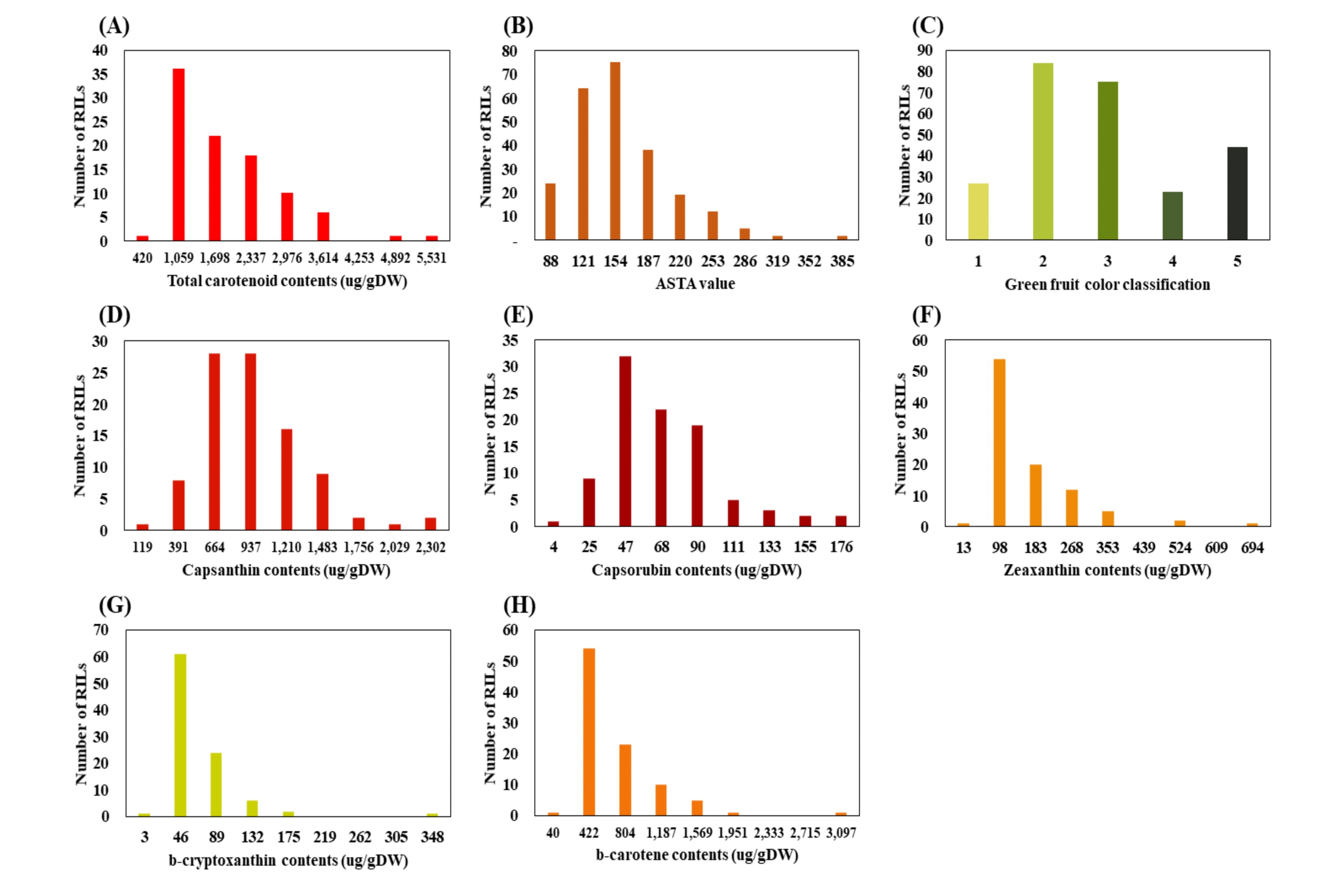

Supplement: Supplementary Figure 3 — Carotenoid pigments distribution in LA RILs. (A) total carotenoid contents (μg/g DW), (B) ASTA value, (C) Green fruit color intensity of green mature fruit, (D) Capsanthin contents (μg/g DW), (E) Capsorubin contents (μg/g DW), (F) Zeaxanthin contents (μg/g DW), (G) β-cryptoxanthin contents (μg/g DW), (H) β-carotene contents (μg/g DW). (A,D–H) Carotenoid contents were measured in 96 RILs. (B,C) ASTA value and green fruit color intensity were measured in 254 RILs. [file Image_3.JPEG]

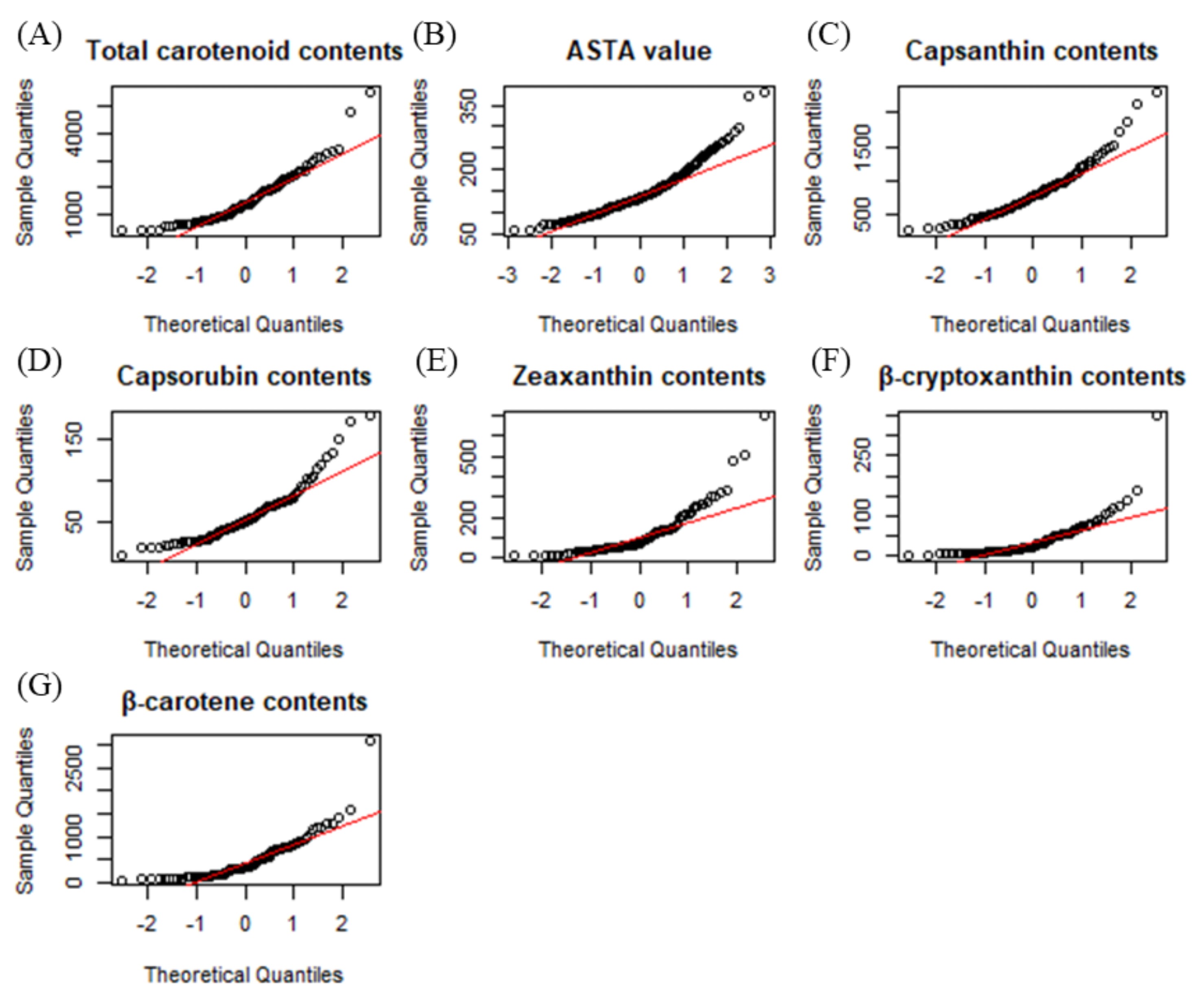

Supplement: Supplementary Figure 4 — Quantile-quantile plot to verify the distribution of carotenoid contents in LA RILs. Q-Q plots of (A) total carotenoid contents, (B) ASTA value, (C) capsanthin, (D) capsorubin, (E) zeaxanthin, (F) β-cryptoxanthin, and (G) β-carotene contents distribution. Red diagonal line in plots represents a theoretical normal distribution. [file Image_4.JPEG]

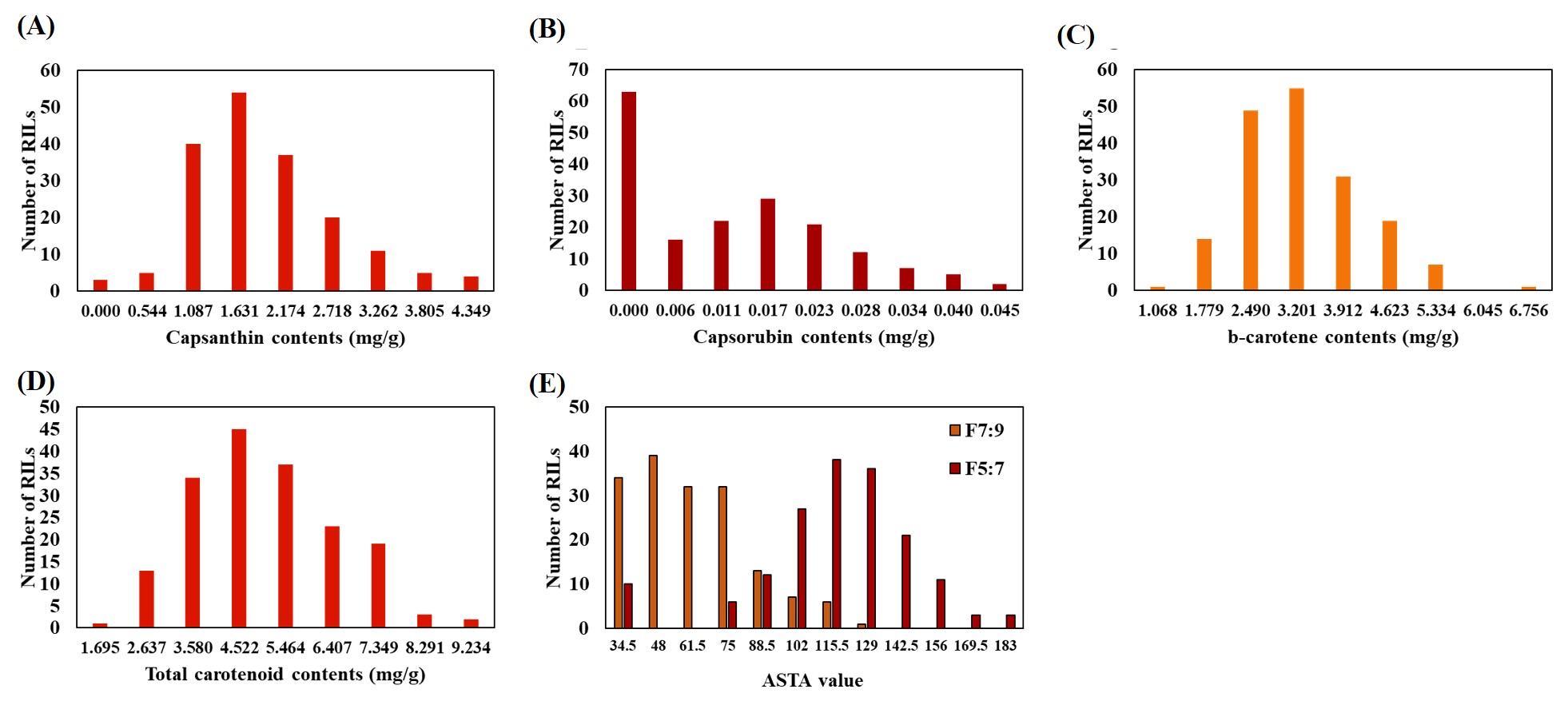

Supplement: Supplementary Figure 5 — Carotenoid pigments distribution in FC RILs. (A) Capsanthin contents, (B) Capsorubin contents, (C) β-carotene contents, and (D) total carotenoid contents (mg/g DW). (A–D) Carotenoid contents were measured in F5:7 generation. (E) ASTA value, ASTA values were analyzed in both F5:7 and F7:9 RILs. [file Image_5.JPEG]

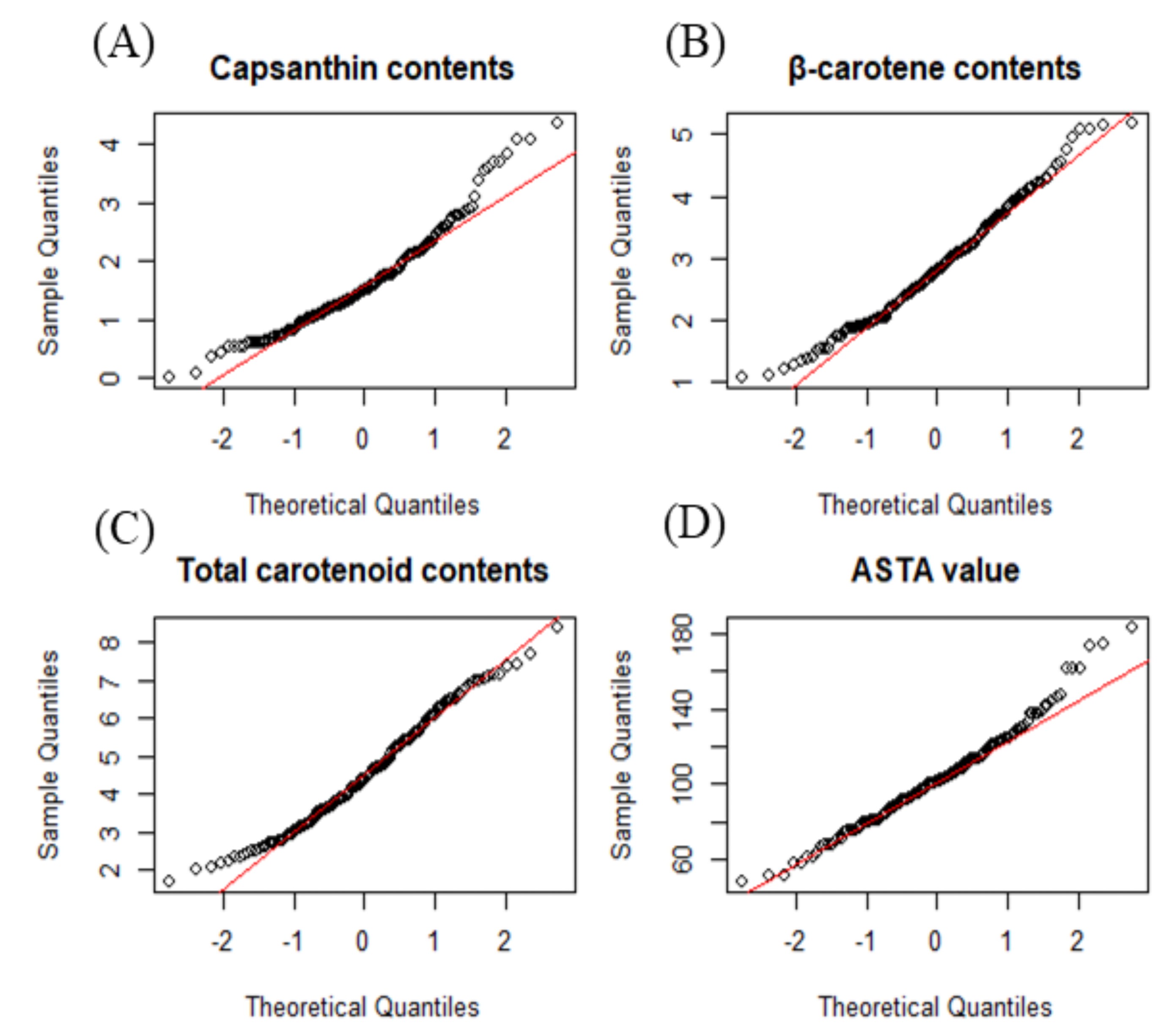

Supplement: Supplementary Figure 6 — Quantile-quantile plot to verify the distribution of carotenoid contents in FC RILs. Q-Q plots of (A) capsanthin, (B) β-carotene, (C) total carotenoid contents, and (D) ASTA value distribution. Red diagonal line in plots represents a theoretical normal distribution. [file Image_6.JPEG]

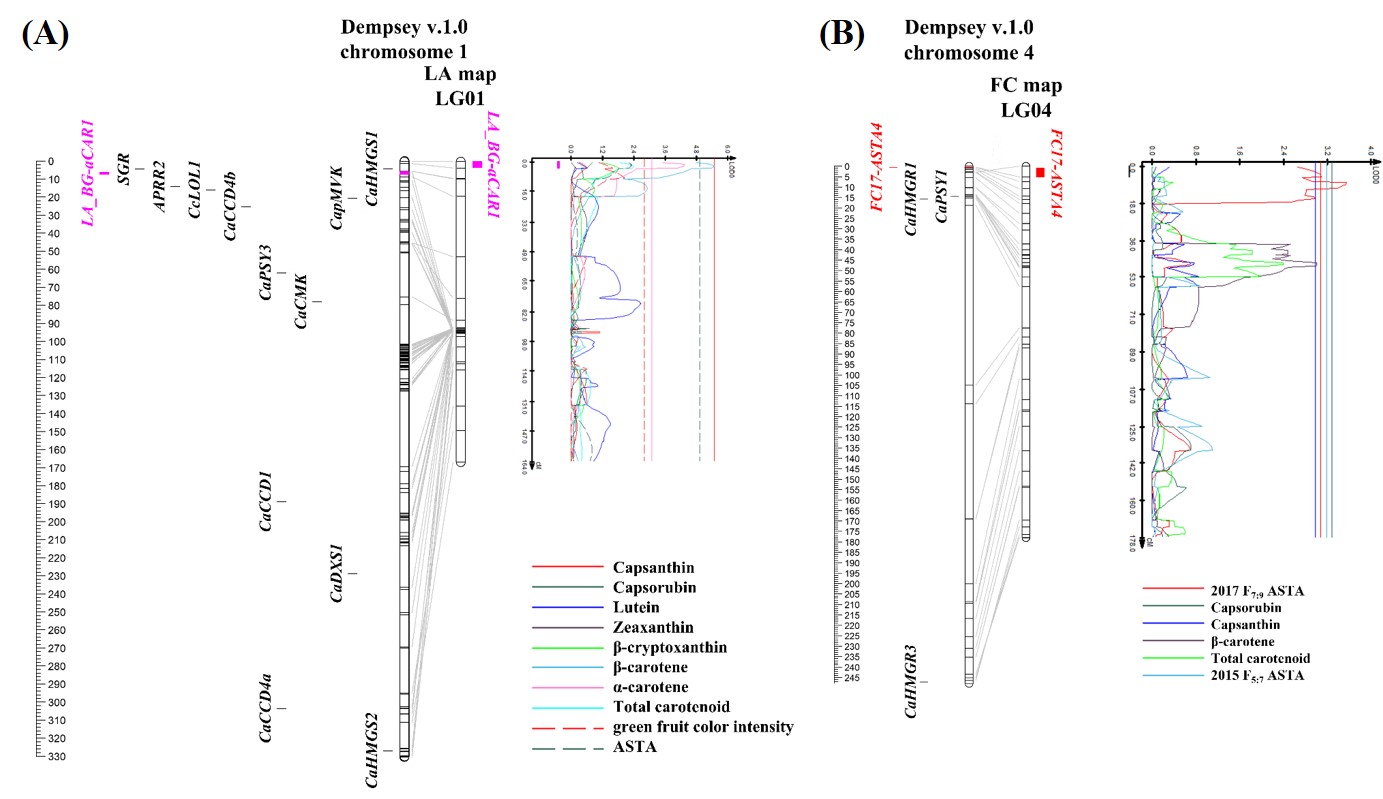

Supplement: Supplementary Figure 7 — Physical map based on Dempsey genome, linkage maps of RILs, and QTL likelihood profile containing the QTL positions. (A) Chromosome 1 and linkage group 1 of LA map. Chromosome 1 is the left bar and linkage group 1 is the right bar. Genes for carotenoid and chlorophyll synthesis are indicated with black boxes and LA_BG-aCAR1 with pink boxes. Graph for LA_BG-aCAR1 is indicated with a pink line. (B) Chromosome 4 and linkage group 4 of FC map. Chromosome 4 is the left bar and linkage group 4 is the right bar. Genes for carotenoid and chlorophyll synthesis are indicated with black boxes and FC17-ASTA4 with red boxes. Graph for FC17-ASTA4 is indicated with a red line. [file Image_7.JPEG]
